# Comprehensive mapping of the *Helicobacter pylori* NikR regulon provides new insights in bacterial nickel responses

**DOI:** 10.1038/srep45458

**Published:** 2017-04-10

**Authors:** Andrea Vannini, Eva Pinatel, Paolo Emidio Costantini, Simone Pelliciari, Davide Roncarati, Simone Puccio, Gianluca De Bellis, Clelia Peano, Alberto Danielli

**Affiliations:** 1 FaBiT - Department of Pharmacy and Biotechnology, University of Bologna, 40126, Bologna, Italy; 2 DIMES Department of Experimental, Diagnostic and Specialty Medicine, University of Bologna, 40138 Bologna, Italy; 3 Institute of Biomedical Technologies, National Research Council, 20090 Segrate, Milan, Italy; 4 Doctoral School of Molecular and Translational Medicine, University of Milan, 20090 Segrate, Italy

## Abstract

Nickel homeostasis is important for pathogenic and ureolytic bacteria, which use this metal ion as enzymatic cofactor. For example, in the human pathogen *Helicobacter pylori* an optimal balance between nickel uptake and incorporation in metallo-enzymes is fundamental for colonization of the host. Nickel is also used as cofactor to modulate DNA binding of the NikR regulator, which controls transcription of genes involved in nickel trafficking or infection in many bacteria. Accordingly, there is much interest in a systematic characterization of NikR regulation. Herein we use *H. pylori* as a model to integrate RNA-seq and ChIP-seq data demonstrating that NikR not only regulates metal-ion transporters but also virulence factors, non-coding RNAs, as well as toxin-antitoxin systems in response to nickel stimulation. Altogether, results provide new insights into the pathobiology of *H. pylori* and contribute to understand the responses to nickel in other bacteria.

Nickel homeostasis is of primary importance for many organisms and especially for pathogenic and ureolytic bacteria, which use this metal ion as enzymatic cofactor for the catalysis of redox reactions and Lewis acid-like functions, with important medical, agricultural and biotechnological implications^1^. The human pathogen *H. pylori* is a paradigmatic example, since its survival in the stomach relies on the catalytic activity of the two nickel-dependent metalloenzymes urease and hydrogenase, respectively involved in acid acclimation and energy metabolism of the bacterium^2^,^3^. Both activities are important for the colonization of the gastric epithelium, leading to long-term infections that correlate with many gastric diseases, including gastritis, peptic ulcers, gastric carcinoma and MALT lymphoma^4^. On the other hand, an excess of nickel ions can be noxious, poisoning other metallo-enzymes or producing reactive oxygen species (ROS)^5^. Nickel-utilizing bacteria must therefore maintain an optimal homeostasis of nickel ions, tightly controlling the balance between their uptake and incorporation in metallo-enzymes or storage proteins. One of the main regulatory factors of nickel homeostasis is the NikR protein, a ribbon-helix-helix (RHH) transcriptional regulator, whose orthologues are present in almost all the main bacterial and archeal clades^6^,^7^. Despite its widespread conservation, NikR regulation has been approached principally in *H. pylori* and *E. coli*, mainly through transcriptional analysis^8^,^9^ and *in vitro* protein-DNA binding studies^10^,^11^,^12^,^13^, leading to the characterization of several bona-fide regulatory targets^14^. While the EcNikR protein functions strictly as a nickel-dependent transcriptional repressor, HpNikR has been proposed to be a more versatile regulator, either inducing or repressing the transcription of a larger cohort of nickel-responsive genes^9^,^11^,^15^. However many studies focused on the regulation of already characterized metal-binding proteins, leaving a systematic characterization on NikR regulation unexplored^13^,^16^,^17^. In this work we sought to fill this gap, combining RNA-sequencing and ChIP-sequencing approaches to provide for the first time the comprehensive mapping of a bacterial nickel-responsive regulon.

## Results

### RNA–seq analysis determines NikR-dependent and nickel–responsive transcriptomes

**Figure 1:**
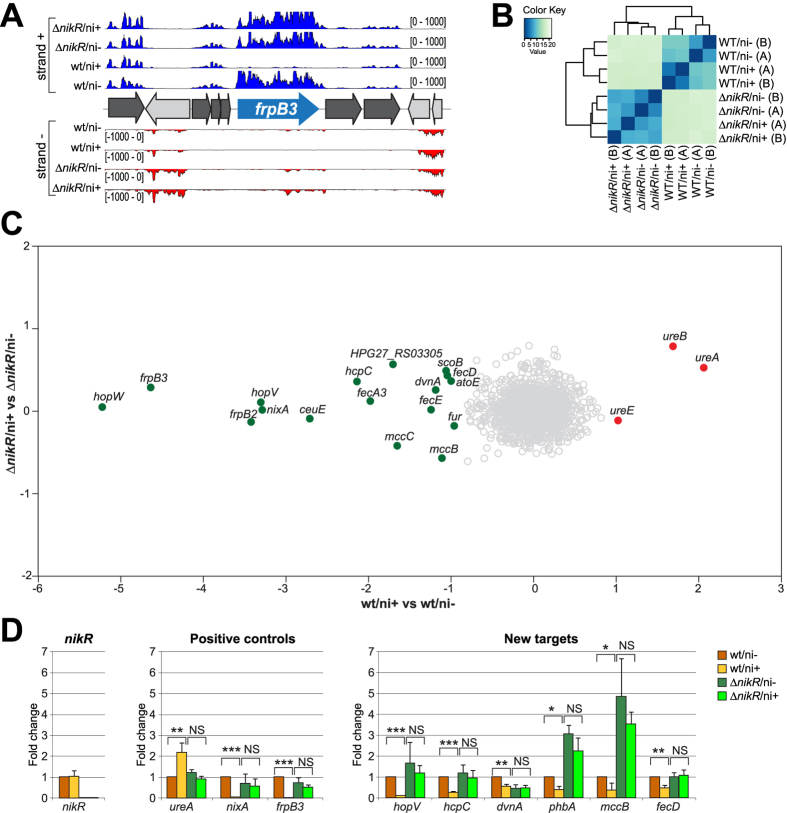
Results of the RNA-sequencing experiment. (**A**) RNA-seq profiles of the *frpB3* genomic locus: wt/ni+, wt/ni−, ∆*nikR*/ni+ and ∆*nikR*/ni− strand specific coverages are shown in blue (strand+) and red (strand−). (**B**) Heatmap showing the Euclidean distances between samples and replicas as calculated from the regularized log transformation performed by DESeq2 on raw counts. (**C**) Effect of nickel treatment on *H. pylori* gene expression: log2FC values of nickel treated (ni+) vs untreated (ni−) samples are reported for wt (x-axis) and ∆*nikR* (y-axis) genotypes. DEGs induced and repressed after nickel addition are represented as filled red and green circles respectively (log2FC ≥ |1|; adj *p* < 0.01); empty grey circles correspond to non differential genes. (**D**) RT-qPCR validation of *H. pylori* nickel responsive genes expression. *nikR* (control), *ureA, nixA, frpB3* (positive controls) and *hopV, hcpC, dvnA, phbA, mccB* and *fecD* (new targets) gene expression levels are reported for wt and ∆*nikR* strain in both ni+ and ni− conditions. Results show fold changes (mean ± SD) relative to the wt/ni− condition. ΔCts were obtained after normalization on 16S rRNA levels; at least three biological replicates were used for the analysis. SD = standard deviation. Ct = threshold cycle. Statistical significance is calculated using the t-test; *p < 0.05; **p < 0.01; ***p < 0.001.

To elucidate the involvement of NikR in the nickel response of *H. pylori*, whole transcriptome analyses were performed by strand-specific RNA sequencing (Fig. S1). To this purpose, a set of nickel treated (ni+) or untreated (ni−) cultures of the wild type G27 strain and of a Δ*nikR* mutant grown to OD_600_ 1.0–1.1 were used as starting material to produce strand specific RNA sequencing libraries. A minimum of 3 Millions of reads were obtained for each sample and for each of the two replicates (Bioproject: PRJNA313048), providing an optimal coverage of the transcripts (Supplementary Table S1). The reliability of the RNA-seq experiment is exemplified by the *frpB3* genomic locus (Fig. 1A), which evidences a clear match between the strand-specificity of the RNA-seq tracks and the annotated CDS, along with the reduction of the signal in the wt/ni+ tracks, corresponding to the reported repression of the *frpB3* gene in response to nickel excess^17^. Samples clustering clearly shows the reproducibility of replicates (average Pearson correlation value on normalized counts: 0.98) and their grouping according to genotypes and/or treatments (Fig. 1B).

Nickel treatment in a wild type (wt) background elicited a total of 20 differentially expressed genes (DEGs; log2FC ≥ |1|, adj p < 0.01), mapping to 14 transcriptional units (Supplementary Table S2 and Fig. 1C x-axis). We observed a transcriptional down-regulation for previously characterized targets of negative NikR regulation such as *nixA, frpB2, frpB3, fecA3, fur* and *ceuE*. We also recorded the significant induction of only three genes, all belonging to the nickel-responsive urease (Fig. 1C). In addition, the RNA–seq analysis pinpointed 11 novel nickel-repressed genes, including several interesting genes coding for membrane-associated proteins and transporters (Supplementary Table S2, Fig. 1C and D), and also a polycistronic operon carried on the pHPG27 plasmid (HPG27_RS07995/HPG27_RS08000; *mccC*/*mccB*). Oddly, we were not able to detect significant variations in the transcript levels of HPG27_RS07055* (*hpn*), HPG27_RS07080* (*hpn2*), HPG27_RS03075 (*hydB*), HPG27_RS00075 (*groES*) and HPG27_RS06735 (*exbB*), which were previously reported to be regulated by nickel or NikR^9^, nor the auto-repression of *nikR* in response to nickel excess^8^,^9^,^18^. When the same comparative analysis (ni+ vs ni−) was performed in the Δ*nikR* strain no genes were differentially expressed upon nickel treatment (Fig. 1C, y-axis), strongly suggesting that the nickel-dependent responses observed in the wt strain are mediated by the *nikR* gene product. These results were independently validated by qRT–PCR on a panel of 9 nickel–regulated genes (Fig. 1D), measuring their expression levels with and without nickel stimulation both in wt and in Δ*nikR* strains. For all these genes, nickel dependent regulation was lost in the Δ*nikR* mutant. In some cases we observed a de-repression corresponding to transcript levels measured in the wt/ni− condition (*ureA, nixA, frpB3, hopV, hcpC* and *fecD*), while in other cases a general decrease (*dvnA*) or increase (*phbA* and *mccB*) in mRNA levels was recorded, suggesting the integration with other layers of transcriptional control (e.g. Fur- or growth phase- dependent regulation, see below). The putative NikR gene targets *hpn* and *hpn2*, which did not change expression levels in all the four conditions analyzed in RNA-seq, were also included in the qRT-PCR analysis (Fig. S4A). Their lack of responsiveness to nickel and *nikR* deletion was confirmed, provisionally indicating that these two genes are not transcriptionally regulated by NikR under the tested conditions.

On the other hand, the comparative analysis between the wt and Δ*nikR* strains under similar nickel stimulation (Δ*nikR*/ni+ vs wt/ni+) outlined 194 DEGs (Supplementary Table S2 and Fig. S2), suggesting that in the Δ*nikR* strain a very large number of genes is deregulated, including many genes not responsive to nickel, likely due to indirect effects. The same comparative analysis in the absence of nickel treatment (Δ*nikR*/ni− vs wt/ni−) supported this interpretation. In fact, out of the 261 DEGs identified, only a handful of nickel-responsive genes pinpointed in the wt strain were spotted, while most differentially expressed transcripts belonged to stationary phase- and/or to the regulon of the Ferric uptake repressor Fur, which is itself part of the NikR regulon^10^ (Supplementary Table S2 and Fig. S2). Thus, the *nikR* deletion has a profound impact on the cell, indirectly affecting the transcription of many genes beyond the relatively tight cohort of nickel-responsive cistrons predicted to belong to its regulon.

### Genome-wide analysis of NikR targets by ChIP-seq

To identify genomic regions bound *in vivo* by NikR, we performed Chromatin Immunoprecipitation assays with a specific NikR polyclonal antiserum (Fig. S3A), followed by deep sequencing (ChIP-seq) in wt and Δ*nikR* strains under nickel-replete (ni+) or untreated conditions (ni−) (Fig. S1). Two sets of biological replicates were employed for the IP analysis, obtaining at least 3 Millions raw reads for each replicate (Bioproject: PRJNA313048). On average more than 98.5% of the reads mapped with good quality on the *H. pylori* G27 reference genome (Supplementary Table 1). The ChIP-seq profiles of the wt strain showed NikR-specific and nickel-dependent enrichments that were absent from the ChIP-seq profiles of the Δ*nikR* mutant and from the control samples (INPUT) obtained by sequencing the sheared chromatin before immunoprecipitation (Fig. S2C; showing the enrichment profiles at the *frpB2* locus). The lack of comparable profiles deriving from immunoprecipitations with antisera specific for other transcriptional regulators demonstrates that the ChIP-enrichment was specific for NikR. Since it is well established that NikR binds specifically to its operator sequences only in presence of the nickel cofactor^19^, we first identified the core of high fidelity NikR target binding sites in the wt strain treated with nickel, and then we analyzed their differential binding respect to wt untreated samples.

**Figure 2:**
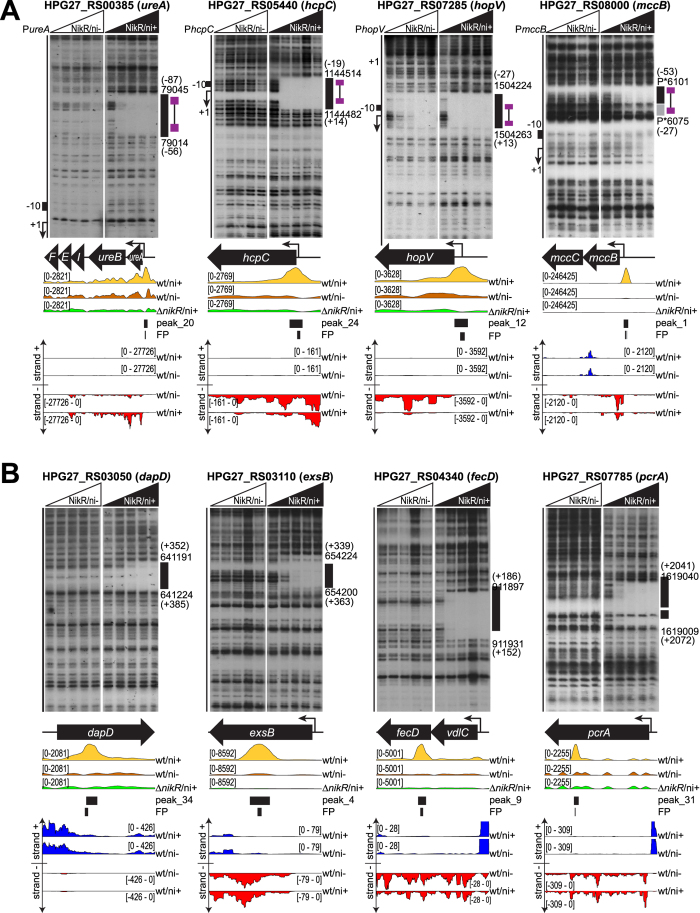
Validation of new NikR promoters and internal peaks by DNase I footprinting. (**A**) Radiolabeled P*ureA*, P*hcpC*, P*mccB* and P*hopV* DNA probes were mixed with 0, 9.7, 29, 97 and 290 nM of the NikR tetramer, without nickel (left side of each panel) or with the addition of 150 μM NiSO_4_ (right side of each panel), before DNase I cleavage. On the right of each autoradiographic film, the G27 genomic coordinates of DNase I protected regions (black boxes) are reported, with position in brackets with respect to the transcriptional start site (TSS). Low affinity binding sites, if present, are shown as grey boxes surrounded by the same information. On the left, a schematic representation of the promoter is provided, with the TSS (+1, bent arrow) and the −10 region (black box). The position of the consensus sequence is reported with violet boxes, corresponding to the two conserved hemi-operator pentamers linked by a black line (15 nt spacer). In the middle panels a scheme of the corresponding transcriptional unit is shown, together with the normalized tag densities obtained from the ChIP-seq experiments (wt/ni+ in yellow, wt/ni− in orange and ∆*nikR*/ni+, negative control in green), the predicted peak extension by Homer2 and the DNaseI protected regions. Representation scales of ChIP-seq tracks are indicated on the left in brackets. In the bottom panels, the RNA-seq strand specific tracks of the corresponding genomic locus are visualized for wt/ni+ and wt/ni− samples (plus strand in blue, minus strand in red). P* indicates coordinates mapping on the pHPG27 plasmid. (**B**) Radiolabeled *dapD, exsB, fecD* and *pcrA* DNA probes were mixed with 0, 9.7, 29, 97 and 290 nM of NikR tetramers, without nickel (left side of each panel) or with the addition of 150 μM NiSO_4_ (right side of each panel). The same elements and information are reported as in panel A.

The peak regions were identified by comparing the ChIP-seq profiles of nickel-treated wt strain (wt/ni+) with those of the nickel-treated Δ*nikR* mutant (Δ*nikR*/ni+), setting the latter as negative control (background) of the whole experiment. Irreproducible Discovery Rate (IDR) analysis outlined the good reproducibility of the replicates (Supplementary Table S1). Consequently, an optimal set of 72 high-quality peaks was defined (Supplementary Table S3). These were mapped with respect to the list of putative *H. pylori* TSSs, obtained by remapping the *H. pylori* 26695 primary transcriptome annotation^20^ onto the *H. pylori* G27 reference genome, or by de novo 5′-end mapping primer extension analysis. 23 peaks were classified as bona-fide “promoter peaks” because they were centered between position −150/+30 with respect to a TSS. The remaining peaks were subdivided into “intragenic peaks” (41 peaks) and “intergenic peaks” (8 peaks) according to the position of their center respectively within or outside the annotated genes. Consistently, many “promoter peaks” overlap the promoters of known NikR–regulated operons. Moreover, we identified peaks on the promoters of the newly identified nickel–responsive operons: *hopV, hopW, hcpC, dvnA* and *mccB*. No peaks mapping to the promoters of *hpn, hpn2, hydA* and *groES* genes were detected (Fig. 2A, Figs S2 and S3).

After the definition of high fidelity NikR binding sites, we set out to determine their nickel-dependence by comparing the read coverage of the 72 high-quality binding peaks in the nickel-treated wt strain (wt/ni+) with the read coverage at the same positions of the untreated culture (wt/ni−). 46 out of the 72 peaks resulted significantly enriched (log2FC > |1|, adj p–value < 0.01), suggesting a differential increase of NikR binding affinity to these targets in the presence of nickel (Supplementary Table S3). The remaining 26 peaks (36%) proved to be not differentially bound, suggesting that NikR binding to these positions is already saturated at the low nickel concentrations provided by the culture medium (Brucella Broth contains 0.2 μM nickel ions)^15^, or that these targets represent nickel-independent binding sites and/or derive from local flaws in ChIP-seq sensitivity.

### Systematic definition of the NikR regulon by RNA-seq and ChIP-seq data integration

RNA-seq analysis identified 20 DEGs responding to nickel in a NikR-dependent fashion. These 20 genes belong to 14 different operons and among them, the urease operon was the only one to be up-regulated. On the other hand, ChIP-seq analysis revealed the presence of 46 NikR binding sites specifically enriched upon nickel stimulation. 19 of these binding sites are located at promoter regions, 10 of them map inside the promoters controlling operons which contain 14 of the 20 previously described DEGs, and one falls between *nikR* and *exbB2* promoters (Supplementary Table S3). The peak on the *fur* promoter was detected but not differentially bound by NikR, suggesting that the regulator binds at this promoter in both high (ni+) and low nickel conditions (ni−). The remaining 5 genes deregulated in the RNA-seq experiment, but not directly associated to a called peak, belong to the operons led by the *nixA, phbA* and *vdlC* genes. Eventually, their ChIP-seq profiles were manually inspected, revealing three detectable peaks (all slightly below the threshold imposed for statistical significance) on the promoter regions. Furthermore for one of them, located on the *nixA* promoter, the direct NikR binding was previously demonstrated^11^. To confirm NikR binding on the nickel responsive operons identified by RNA-seq analysis, we performed *in vitro* DNaseI footprinting assays with purified recombinant NikR protein (Fig. 2A and Fig. S3). Remarkably, all the probes exhibited a detectable footprint of 32 bp (average size) starting at the minimal concentration of NikR used. Protection appeared only in presence of nickel, with the exception of *hopV* and *hopW* promoter regions for which *in vitro* binding was observed also in the absence of nickel, although the protection was remarkably stronger in the presence of the metal co-factor. For *hopV, hopW, hcpC, vdlC, dvnA* and *mccB* the NikR element overlapped the TSS and/or the core promoter region, while binding sites farther upstream (>50 bp from TSS) were verified only for *ureA* and *phbA*. Finally, 9 out of 19 promoter peaks individuated by ChIP-seq analysis that were differentially enriched after nickel stimulation, were not associated with differential nickel regulation in RNA-seq, suggesting that nickel-responsive binding of NikR to these regions does not result in measurable transcriptional effects, at the conditions used.

### Extensive intragenic binding of NikR

A remarkable number of predicted NikR binding sites mapped in intragenic positions (41/72) and 23 of them were differentially enriched upon nickel stimulation. To exclude the possibility that this result derived from a bias in the ChIP experiment, footprinting assays were performed on a panel of intragenic differentially enriched binding sites (Fig. 2B). In all cases NikR exerted a clear and nickel-dependent protection centered within the called peak, validating ChIP-seq results and revealing extensive intragenic NikR binding not previously reported. Similarly to the promoter peaks not associated with nickel dependent transcriptional regulation, also the intragenic NikR targets were not affected by transcript levels variations (Fig. 2B), suggesting that more than 70% of NikR *in vivo* targets are orphan in terms of regulatory control. As such, the role of the transcriptionally orphan NikR binding sites may envisage also putative nucleoid-associated functions and/or (long-range) effects on gene expression, which certainly deserve further investigation in the future.

### Three ncRNAs belong to the NikR regulon

**Figure 3:**
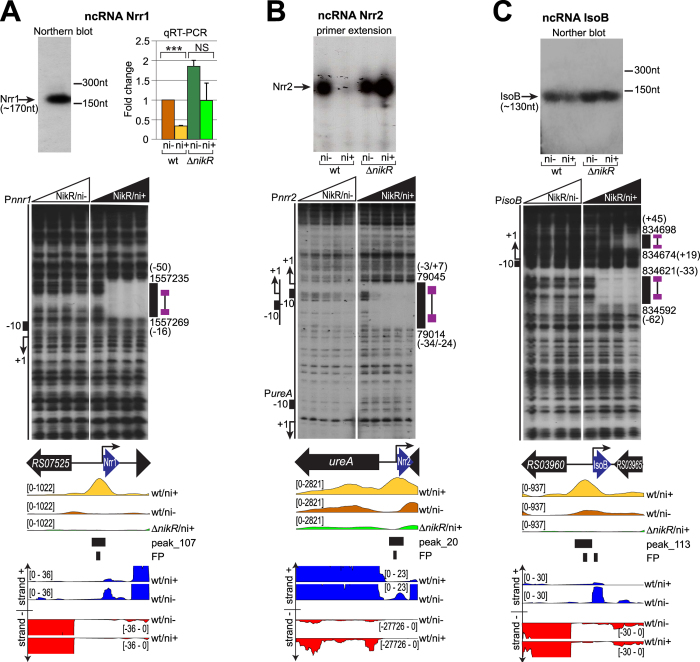
Validation of the new NikR-dependent nickel-regulated ncRNAs. Top panels: transcriptional analysis of ncRNAs in wt/ni−, wt/ni+, ∆*nikR*/ni− and ∆*nikR*/ni+ conditions. (**A**) Northern blot of Nrr1 (left) and quantitative RT-qPCR of its transcript levels (right) (see legend 2D for details). (**B**) Primer extension analysis of the Nrr2 transcript. (**C**) Northern blot of the IsoB transcript. Middle panels: DNase I footprinting of radiolabeled Nrr1 (**A**), Nrr2 (**B**) and IsoB (**C**) DNA probes, mixed with 0, 9.7 (only panel B), 29, 97 and 290 nM of the NikR tetramer, without nickel (left side of each panel) or with the addition of 150 μM NiSO_4_ (right side of each panel). Uncropped blots and gels are provided in the Supplementary Information. Legends and symbols as in Fig. 2.

Interestingly, the ChIP-seq analysis mapped a NikR binding site to the promoter of a transcript corresponding to *aapA6* (HPnc8050) in strain 26695^20^. This gene encodes a small ORF-encoding component of a putative class I toxin-antitoxin system. Analysis of the RNA-seq tracks showed a transcript downstream of this putative promoter also in strain G27: the TSS was validated by primer extension analysis, while RNA-blotting showed a single product of 170 nt (Fig. 3A), with only partial sequence similarity to the 5′ region of the *aapA6* transcript. In fact, the transcript encoded by the G27 strain lacked any obvious ORF and no antisense transcript was detected on the opposite strand (Fig. 3A). These observations suggest that in strain G27 this locus encodes a non-coding RNA rather than a toxin-antitoxin system. The transcript was accordingly renamed Nrr1, for NikR-regulated sRNA1. The binding of NikR to the *nrr1* promoter was confirmed *in vitro* by DNase I footprinting, with a strong protection occurring at the minimal concentration of protein tested only with the presence of nickel (Fig. 3A). Nrr1 expression levels were assayed by qRT-PCR, with results showing reduced transcript levels in response to nickel, and the loss of regulation in a Δ*nikR* genetic background, suggesting that NikR represses Nrr1 under nickel-replete conditions (Fig. 3A).

Another interesting case is represented by the intergenic region upstream of *ureA*, encompassing a divergent TSS, conserved in strain G27 and 26695^20^. RNA-seq data showed strong nickel and NikR-dependent down-regulation of this transcript (Fig. 3B), that corresponds to the conserved ncRNA HPnc0260. Direct NikR binding within the core promoter region was further validated by DNase I footprinting, demonstrating that a single NikR operator oppositely regulates the divergent *ureA*-HPnc0260 promoters (Fig. 3B), repressing the HPnc0260 ncRNA (renamed Nrr2: NikR-regulated sRNA2) while inducing the transcription of the urease operon.

Refinement of the ncRNA annotation also showed that a peak on the HPG27_RS03960 gene encompassed the promoter of a divergent transcript, homologous to the small ORF-encoding mRNA/antisense RNA family *aapB*/IsoB (HPnc4170 and HPnc4160). AapB-IsoB together were proposed to form one of several class I toxin–antitoxin loci, in which the Iso transcript functions as asRNA antitoxin to modulate the expression of the (sense) *aap* transcript^20^. While the IsoB antisense transcript was confirmed by primer extension analysis (not shown) and Northern blot analysis (Fig. 3C), the *aapB* transcript was barely detectable in the G27 transcriptome (Fig. 3C), and we were not able to confirm its expression with alternative techniques nor in different growth conditions (data not shown). Thus, IsoB may be the unique transcript arising from this locus, embodying a ncRNA that could target other mRNAs or Iso-Aap families in trans. Direct binding of NikR to the IsoB promoter was validated by footprinting analysis (Fig. 3C). Moreover, the IsoB transcript was down-regulated by nickel, while in the Δ*nikR* mutant the response to nickel vanished (Fig. 3C), strongly suggesting that, similarly to Nrr1 and Nrr2, IsoB is repressed by NikR. In conclusion, the integration of different genome-wide approaches permitted the identification of three new non coding transcripts repressed by NikR in nickel-replete conditions, suggesting that the responses to nickel may include extensive post-transcriptional regulatory events, which could explain some of the pleiotropic effects observed in the *nikR* knockout mutant (see also Fig. S2).

### NikR consensus sequence

**Figure 4:**
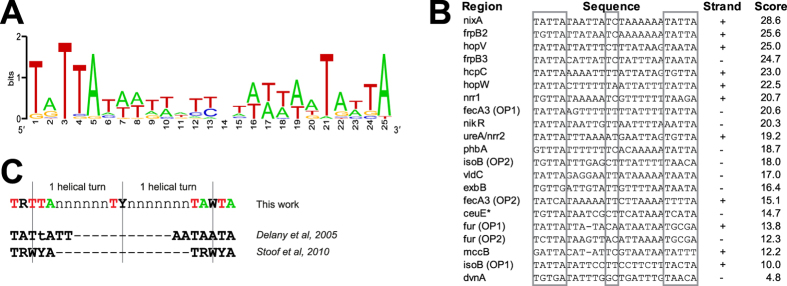
NikR consensus sequence. (**A**) Weblogo of the NikR consensus sequence elaborated by GLAM2 considering all validated NikR operators within gene promoters. (**B**) List of the DNA sequences aligned by GLAM2 to generate the consensus sequence, with the strand used for the alignment and the scores resulting from re-alignement of each sequence to the consensus. Homologous regions in G27 were used if the operator was originally characterized in a different strain. *The NikR operator on P*ceuE* was re-mapped accordingly to the Maxam–Gilbert G + A reaction reported in ref. 14. (**C**) Proposed NikR consensus sequence and comparison with the published ones.

Finally, we used GLAM2^21^, to investigate the consensus for NikR binding (Fig. 4A). Employing the promoter sequences protected by NikR in the footprinting experiments (Fig. 4B, this work and previously reported sequences^10^,^11^,^13^,^16^), we obtained the conserved pentamers TRTTA and TAWTA, positioned 15 nt apart from each other, with a relevant but less conserved TY element in between (Fig. 4A). This consensus sequence closely matches a TRWYA dyad motif predicted by bioinformatic analyses^22^. The 20 nt distance between the center of the 2 pentamers is in accordance with binding of one NikR tetramer to two hemi-operator regions separated by exactly two DNA helix turns^23^,^24^ (Fig. 4C). Moreover, the two half-sites of the consensus motif appear to be almost completely conserved among different *H. pylori* strains, hinting at a conserved operator readout mechanism (Supplementary Table S5). Interestingly, the first thymine of the second repeat is highly conserved among the NikR-bound promoters, since all the sequences used to generate the consensus have a T in that position, with the exception of P*nikR,* bound by NikR with lower affinity^12^ (see also Supplemenatry Table S6). Previous analysis of the sequence determinants for a tight DNA-protein binding has identified this position as an essential element for a low *K*_D_^25^. Coherently, the operators encompassing a thymine in this position, exhibit high binding affinity of NikR in our footprinting experiments (Supplementary Table S6). On the contrary, the presence of a cytosine characterizing one of the hemi-operator motifs of the 26695 *ureA* operator^25^, appears not to be a pre-requisite for high affinity binding of NikR.

### NikR regulates two *hop* paralogues encoding predicted outer membrane transporters

**Figure 5:**
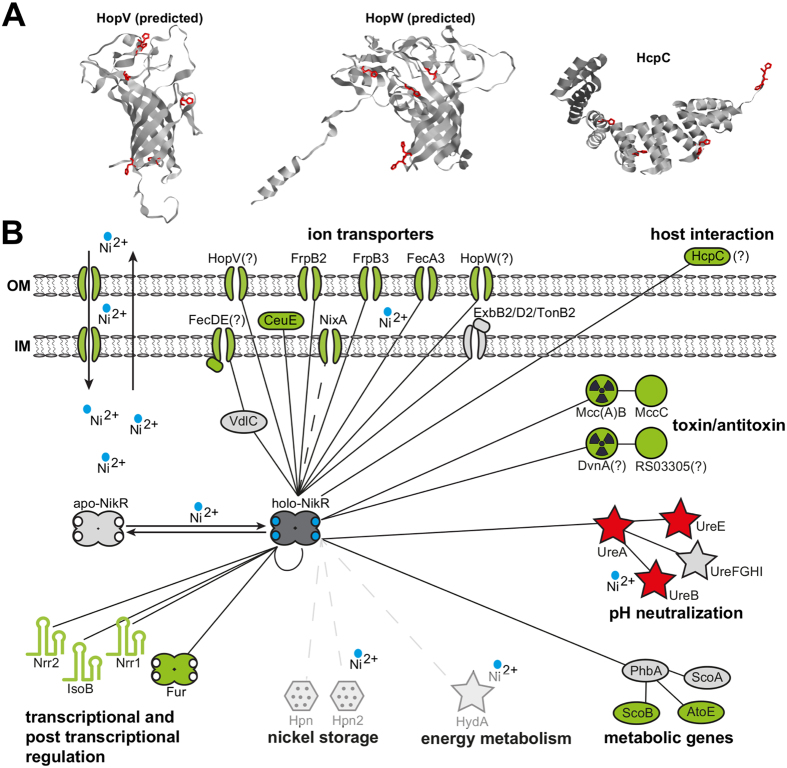
Model of the NikR regulon. (**A**) Predicted structures of HopV and HopW proteins, computed by the online tool Phyre2 and deposited crystallographic structure of the HcpC protein (PDB 1OUV). Histidine residues are highlighted in red. (**B**) The network connects NikR to the first gene of each transcriptional unit (TU) under direct (validated by footprinting) transcriptional control. Filled lines indicate interactions verified in this study, while dotted lines denote uncertain interactions. Genes part of the same TU are linked to the first gene of the cistron, with red, green and grey colours symbolizing induction, repression or absence of NikR-dependent regulation, respectively. Symbols are related to the predicted biological function.

Our analysis also indicated that the *hopV* and *hopW* genes, encoding *Helicobacter*-specific outer membrane proteins, predicted to function as low-permeable broad range porins or as specific transporters for yet undetermined ions^26^, are directly regulated by NikR (Supplementary Table S3 and Fig. 5). Comparative genomic analysis with Ortholuge, a computational method that can generate precise orthologue predictions between species on a genome-wide scale^27^, indicated that *hopV* and *hopW* are conserved in gastric *Helicobacter* species, e.g. *H. acinonychis* and *H. cetorum*, while they are not conserved in non-gastric *Helicobacter* lineages. This prompts the hypothesis that these genes may have a role in nickel homeostasis, contributing to the adaptation to the gastric niche. Tertiary structure prediction indicated a transmembrane β-barrel fold for both *H. pylori* HopV and HopW, with histidine residues exposed on both surfaces as well as internal to the channel, tentatively suggesting a function as metal ion channels across the outer membrane (Fig. 5A). Interestingly, a *hopV* knockout mutant, showed a significant derepression of NikR regulatory targets under physiological growth conditions (Fig. S5), indicating lower intracellular concentration of nickel ions. This indirect evidence suggests that HopV may be involved in the trafficking of nickel ions.

## Discussion

Much of our knowledge on nickel-dependent transcriptional responses derives from the detailed study of single genes or gene clusters controlled by the NikR family of regulators in different bacteria. These studies were generally biased towards nickel- or metal-binding proteins and transporters, providing a patchy view on NikR regulation. On the other hand, HpNikR is the only member of the NikR transcription factor family that has been addressed by genome-wide studies to date^8^,^13^. Our results indicate that in *H. pylori* NikR regulates 17 nickel-responsive transcriptional units, mainly repressed by the regulator, representing a fairly compact regulon with clearly defined functional roles (Fig. 5). This sets NikR as specific transcription factor dedicated to nickel-dependent responses rather than a pleiotropic regulator, despite other RHH-type regulators have been shown to act as global transcriptional regulators (e.g. *Pseudomonas* AmrZ^28^). In fact, considering the hundreds of DEGs indirectly affected by the *nikR* deletion, we can infer that some predicted targets validated by *nikR* mutational analysis reflect a pleiotropic adaptation to stresses deriving from the altered physiology and/or pervasive changes in the transcriptome of Δ*nikR* strains. One likely explanation is that together with the deregulation of the Fur regulon this effect arises from the deregulation of the NikR-dependent small ncRNAs, which may post-transcriptionally control the output of the nickel regulatory responses.

Interestingly, the NikR regulon includes both genes specific to the *Helicobacter* lineage as well as genes that are conserved in other bacteria. For example, the *fecA, frpB* and *tonB-exbBD* metal transporter systems, together with urease and urease chaperones, are widespread in Gram-negative organisms and have been repeatedly reported to respond to nickel^29^,^30^. In addition our analysis has included the *vdlC*-*fecD*-*fecE* operon in the NikR regulon. This operon comprises the *fecD* and *fecE* genes, that are annotated as putative components of an ABC transporter for iron (III) dicitrate. Nevertheless, their expression proved independent from iron stimulation^31^ and resulted to be regulated by NikR in response to nickel availability (Fig. S3). Moreover, the inactivation of *fecD* in *H. mustelae* showed reduced uptake of nickel and cobalt ions, along with reduced urease activity, indicative of a low availability of intracellular nickel, while no effect on iron trafficking was detected^22^. These observations indicate that FecD orthologues may act as nickel trafficking systems.

NikR also appears to regulate toxin-antoxin systems that are conserved in other bacterial species. For example, the *mccB* and *mccC* genes carried by the pHPG27 plasmid share similarity with the *E. coli mccB* and *mccC* genes, involved in the production of microcin C (McC). McC is a 1.2 kDa antibiotic peptide produced by many strains of the *Enterobacteriaceae* family, which inhibits the growth of phylogenetically-related species by interfering with the translation machinery, if the corresponding antitoxin is not expressed^32^. In *E. coli* the MccA pre-toxin is adenylated by MccB into the mildly active McC toxin intermediate^33^, that is eventually exported by the MccC protein, protecting the producer organism from their toxic effect. Recent findings indicate that the *H. pylori* strains containing this system are able to produce and export at least the intermediate form of McC^34^. These results suggest a strategy for rebuffing other microorganisms or cognate *H. pylori* strains lacking the episomal element, in order to succeed in the struggle for nickel acquisition. Similarly, the HPG27_RS03310-HPG27_RS03305 bicistronic operon appears to encode a nickel-responsive chromosomal antimicrobial system. In fact, HPG27_RS03310 exhibits 69% similarity with the *dvnA* gene of *Carnobacterium divergens*, encoding a class IIc Sec-secreted bacteriocin Divergicin A precursor^35^. It also shares high similarity to enterocin-B and enterocin-Q, the other two components of the class IIc bacteriocins. In *C. divergens* the *dvnA* cistron is followed by the *dviA* gene, encoding a transmembrane protein protecting the cell from the negative effect of the DvnA toxin^36^. In *H. pylori* the downstream gene in the operon (HPG27_RS03305) also encodes a membrane protein, that clusters together with HPG27_RS03310 in more than 50 different strains of *H. pylori* and *H. acinonychis*. Together, these findings represent the first evidence of nickel-responsive toxin-antitoxin systems, pointing at a possible selective antimicrobial weapon induced by nickel starvation, which may confer a selective advantage in conditions in which nickel represents a limiting source.

On the other hand, several of the NikR regulon members coding for membrane-associated or exposed proteins seem to be specific to *Helicobacter*. For example, in addition to the *hopV* and *hopW* OMP paralogues described before, the *hcpC* gene encodes for a secreted and OM-absorbed protein, which belongs to the family of *Helicobacter* cysteine-rich proteins^37^. HcpC is predicted to fold into a soluble α-helix-rich structure, with few histidines and a high number of exposed cysteine residues, hinting at a possible role in binding soluble metal ions (Fig. 5A). Members of the Hcp family have a poorly characterized putative β-lactamase function. Moreover, their expression associates with chronic atrophic gastritis^38^, and they have been shown to interact with host cells, inducing the production of IFNγ and other proinflammatory cytokines^39^. HcpC in particular fulfills different roles in the *H. pylori* virulence arsenal, ranging from immunoevasion to control of host invasion^40^. Evidence that *hcpC* is repressed by NikR and nickel, suggests that under nickel starvation the bacterium may boost its virulence, through one or more of the HcpC functions.

Our analysis also demonstrates that HpNikR, similarly to EcNikR, functions principally as repressor of transcription, with the only exception represented by the apparently positive regulation of the urease operon (Supplementary Table S2; see also below). Some of the down-regulated targets identified are well-known genes of the NikR regulon involved in metal trafficking: *nixA, frpB2, frpB3, ceuE, fecA3* and *fur*. Hence, our analysis is in accordance with a significant portion of previous literature^8^,^9^,^11^,^13^,^15^,^16^,^18^,^41^. Moreover, our analysis pinpointed *hopV* and *hopW* to the NikR regulon, validating previous indications^8^, and identified new targets (*hcpC*, [*vdlC*]-*fecD*-*fecE, mccB*-*mccC*, [*phbA*]-*scoB*-*atoE, mccB*-*mccC, dvnA*-*HPG27_RS03305*) and non-coding transcripts (nrr1, nrr2, isoB; see Supplementary Table S6). Other genes that were previously shown to be under the control of NikR were not confirmed in our analysis: *hpn, hpn2, nikR, exbB*-*exbD*-*tonB, hydA* amongst others^8^,^9^. These discrepancies possibly arise from the different experimental set-up: we used a medium supplemented with FBS, which has a higher complexity compared to the more neutral ß-cyclodextrin used in the study of Muller *et al*.^9^. Moreover, while most of the previous investigators treated with low concentrations of nickel for prolonged times (see Supplementary Table S6), we chose a brief impulse of high nickel excess to focus on the nickel- and NikR-dependent responses. This approach likely minimizes the collateral effects of a prolonged exposure to nickel, which could elicit slow occurring adaptive responses through the activation of other pathways: the stress response circuit (*hrcA, hspR, groELS*^8^), the Fur regulon, growth-phase dependent effects and post-transcriptional regulation by non-coding RNAs. Nevertheless, we cannot exclude that our set-up underestimates the cohort of genes transcriptionally controlled by NikR, since the short time frame could be insufficient to detect significant transcriptional variations, in case of highly stable mRNAs and low affinity binding sites.

One particularly intriguing aspect emerging from this study is the absence of regulation of *nikR* in response to nickel, while this gene is widely considered as autorepressed. In fact, the NikR protein is able to bind its own promoter in presence of nickel ions^8^,^10^,^12^,^25^,^42^. Moreover, the expression levels (both mRNA and protein) decrease after prolonged exposure to nickel^8^,^9^,^18^. The divergent *exbB-exbD-tonB* operon is reported to be regulated by NikR similarly to the *nikR* gene: NikR binds to the *exbB* promoter, sharing the P*nikR* operators^10^, and the mRNA levels of the downstream genes are repressed in response to prolonged nickel treatment^9^. In the ChIP-seq experiment we were able to pinpoint a nickel-dependent peak on the *nikR-exbB* intergenic region, which indicates *in vivo* binding of NikR to this locus. However, we were unable to detect a trascriptional response of *nikR* and *exbB-exbD-tonB* operons under conditions promoting the response of the other genes belonging to the NikR regulon. In this respect, it is important to recall that P*nikR* is a weak binding target of NikR, with a 100-fold higher *K*_D_ compared to other promoters^12^. Moreover, nickel-dependent variations of *nikR* mRNA or NikR protein levels were observed only after prolonged exposure to the metal (5 h with 10 μM nickel for mRNA and 10 h for protein)^9^, or were not detectable at all^41^. Similarly, variations of *exbB* transcript levels (and also variations of *hpn, hpn2,* and *hydA* mRNA) were observed only after prolonged treatment with nickel^9^. Because of the low-affinity binding of NikR on P*nikR* and P*exbB* promoters and the slow kinetics of transcriptional response of these genes to nickel treatment, it is possible that NikR binds and/or regulates these promoters only after prolonged nickel exposure. Another possibility is that regulation at these loci occurs through a more complex regualtory mechanism. Interestingly, Fur binds these promoters on two operators which partailly overlap those of NikR^10^. *In vitro*, Fur binding to the latter occurs in absence of metal ions, but increases when metal ions, including nickel, are added^10^. Competitive footprinting experiments also demonstrated that Fur is able to compete for NikR binding on the *nikR* promoter, suggesting that a competition between these two transcriptional factors could occur *in vivo*^10^. Moreover, since Fur is under the transcriptional control of NikR, the effect exerted by NikR on the *nikR* and *exbB* promoters could in principle be also mediated by Fur, resulting in an indirect and slower response kinetics at those promoters. Finally, it is worth noticing that Fur is able to use also nickel as co-factor to bind DNA^10^. As such it may contribute to transduce nickel-dependent responses, but only after prolonged exposure to the metal, since a 20 min nickel pulse is not sufficient to activate the Fur regulon (Fig. 1C). Taken together, our results suggest that the regulation of *nikR, exbB, hpn, hpn2* and other genes with a slow response to nickel may be under the control of a different regulatory mechanism or circuit with respect to the more responsive members of the NikR regulon. The interplay between Fur and NikR seems to have a paramount role in these responses and clearly deserves further investigations.

Another paradigmatic example of the complexity of nickel regulation in *H. pylori* is represented by *hpn* and *hpn2*, which code for two highly expressed histidine-rich paralogues, fundamental for the colonization of the host^43^. Nevertheless, *hpn* and *hpn2* respond to nickel very differently in distinct *H. pylori* strains (Fig. S4). For *hpn* this is likely imputable to significant sequence divergence within the promoter region (Fig. S4C). This observation appears striking, given the evolutionary importance of *hpn* and *hpn2* in the adaptation to the gastric habitat and their essential role for colonization. For example, *hpn2* was induced in response to the short (20 min) stimulation with 500 μM nickel in strain 26695, while we were not able to record any variation of the transcript in strain G27 (Fig. S4A), even though the low ChIP-seq enrichment values and the presence of a low affinity NikR binding site verified by *in vitro* footprinting, are compatible with a weak interaction of NikR with the promoter (Fig. S4B and S4D). Moreover, when the treatment with lower concentrations of nickel (20 μM) was prolonged for 5 hours, a sheer down-regulation of the *hpn2* transcript was detected in strain G27 (Fig. S4A). Notably, these responses were maintained in the *nikR* knockout mutant, suggesting that another regulator participates to the transcriptional response of *hpn2*. Currently, the best candidate appears to be the ferric uptake regulator Fur, which is able to use nickel as co-repressor and bind to the P*hpn2* promoter, repressing its transcription^44^.

Another possible explanation is that the positive effect of NikR may be postranscriptionally mediated by ncRNAs. The observation that positive responses on *hpn, groESL*, and *hydA* were recorded hierarchically after prolonged growth in nickel-replete media^9^, and do not respond promptly to nickel stimulation as all the other repressed targets, supports this hypothesis. Moreover, the *hpn* transcript is subjected to post-transcriptional control by aconitase, a metal-sensor contributing to bacterial pathogenesis^45^. Together with s-SodF, a recently identified sRNA derving form the 3′UTR of a nickel regulated transcript in *Streptomyces coelicolor*^46^, the three ncRNA regulated by NikR reported in this work represent the first examples of nickel-responsive sRNAs. Thus, in analogy to the conservation of iron-regulated small RNAs pioneered by the discovery of *E. coli* RyhB^47^, also nickel-responsive sRNAs maybe widespread and extend their function beyond metal homeostasis, involving the expression of virulence-associated factors in pathogenic bacteria. In this light, the possibility of a nickel-responsive post-transcriptional regulation of urease deserves further investigation, since other sRNA regulating urease have been reported both in *H. pylori*^48^ as well as in enterohemorrhagic *E. coli*^49^. In fact, the evidence that the unique NikR binding site upstream of the urease promoter is clearly associated with nickel-responsive repression of the divergent Nrr2 transcript, poses the question whether NikR binding to this operator also directly activates the *ure* promoter. A recent study suggested that NikR has acquired a RNA polymerase (RNAP) interacting domain^50^ and could therefore act as transcriptional activator. In this scenario, it is surprising to notice that upon the acquisition of a RNAP interacting domain a key transcription factor like NikR is engaged in the control of only one (urease) operon.

Finally, the integration of RNA-seq and ChIP-seq datasets allowed the identification of a large number of genomic loci bound by NikR in response to nickel excess, which are not related to transcriptional regulation. This is not surprising, since for many regulator targets identified by genome-wide location analyses, including those of HpFur^44^, a detectable transcriptional effect on the neighbouring genes is absent when the regulator is depleted^51^. This may be due to several reasons. NikR may be bound upstream of a gene where it has little impact on levels of transcription, because of the overriding influence of another regulatory mechanism or because its role is only to to fine-tune the levels of transcription. While this may be true for complex promoters, e.g. the *arsR* promoter were the interaction of Fur and NikR governs a sofisticated signal integration logic^52^, this possibility does not explain the role of intracistronic binding sites. Tentatively, intragenically bound NikR could hint at nucleoid-associated functions important for chromosome organization^53^. In this respect, the intragenic binding sites could also represent dedicated ‘parking bays’ for the regulator, hold in place by nucleoid topology in order to maximize regulator concentration at a spacially proximal regulatory element. Finally, it has not to be excluded that the intragenic targets may have no direct functional relevance, since bacterial genomes are permissive to transcription factor binding *per se*^54^.

In conclusion, the first comprehensive insight into bacterial nickel-dependent responses discloses a regulon specifically devoted to nickel-trafficking and metallo-enzyme genes, with the potential of extensive post-transcriptional regulatory capabilities, and an intimate interlink with the Fur regulatory circuit. Interestingly the NikR regulon also includes antibiotic and virulence factors not immediately associable with nickel homeostasis, which may contribute to shape responses in a bacterial community. As such the insights on the *H. pylori* NikR regulatory network contribute to a broader understanding of the nickel metabolism in other ureolytic microorganisms colonizing soil or the human oral, gastrointestinal, urinary and respiratory tracts, with evident biotechnological and biomedical implications.

## Materials and Methods

### Bacterial strains and growth conditions

All *H. pylori* strains used are listed in Supplementary Table 4. Bacteria were recovered from frozen glycerol stocks and propagated on BBL Brucella (BD) agar plates containing 5% fetal bovine serum FBS. Bacteria were grown at 37 °C in jars using CampyGen™ (Oxoid) gas–packs or in a water–jacketed thermal incubator (9% CO_2_, 91% air atmosphere, and 95% humidity) for 24–48 hr. Liquid cultures were grown in BBL Brucella Broth (Sigma–Aldrich) supplemented with 5% FBS and Dent’s or Skirrow’s antibiotic supplement at 37 °C in glass flasks with gentle agitation (125 rpm). In order to faithfully record the transcriptional responses to nickel transduced by NikR (DNA-binding) activity, and avoid the bias deriving from slow adaptation to growth in nickel-rich conditions instead, the bacteria were treated for short times with relatively high concentrations of nickel (500 μM Ni^2+^ for 20 min). *E. coli* strains were grown in Luria–Bertani (LB) agar or in LB broth. When required, 100 μg/ml ampicillin was added.

### DNA manipulations

DNA amplification, restriction digestions and ligations were carried out with standard molecular techniques, with enzymes purchased from New England Biolabs.

### RNA preparation, qRT assays, primer extension and Northern blot

Bacterial cultures of wild type and Δ*nikR* strains were grown to OD_600_ of 1.0–1.1 and split into 2 sub–cultures of 5 ml each that were treated either with 500 μM nickel (ni+) or with the same volume of sterile water (ni−) for 20 minutes. Samples were stopped by addition of 625 μl RNA stop solution (95% ethanol, 5% acid phenol) and RNAs were purified using 1 ml of Tri–reagent (Sigma-Aldrich) for each sample, following the manufacturer instructions. RNAs were treated with DNaseI prior to cDNA synthesis^55^. Two μl of 1:10 diluted cDNAs were added to 5 μl of PowerUp™ SYBR^®^ Green Master Mix (TermoFisher) and 400 nM of forward and reverse oligonucleotides in a 10 μl final volume. The qRT–PCR program was performed as previously described^55^. Primer extension analysis was performed using 15 μg of total RNA and 0.1 pmol of radiolabeled probe. Northern blot assay was performed using 17 μg of total RNA and 1.25 pmol of radiolabeled oligo probe.

### Overexpression and purification of recombinant NikR protein

Recombinant NikR protein was overexpressed and purified under native conditions as previously described^19^. The purified protein was dialyzed overnight against NikR footprinting buffer (20 mM Hepes pH 7.85, 50 mM KCl, 0.02% Igepal, 0.1 mM DTT and 10% glycerol) prior to the DNA binding experiments. A Bradford colorimetric assay (Bio–Rad) was used to quantify the protein fractions with bovine serum albumin as standard.

### Preparation of a polyclonal α-NikR antibody

The α–NikR antisera were generated by immunizing rabbits with affinity–purified recombinant NikR protein (without tag) by Biotem Custom Antibodies and Services. After the final bleed, the antisera were purified by 3 sequential precipitations with 35% saturated (NH_4_)_2_SO_4_ and subsequent dissolution in water. The partially chemical purified α–NikR antibody was assayed by western blot analysis on *H. pylori* total extracts, showing a single major band corresponding to the expect molecular weight of NikR.

### Chromatin Immunoprecipitation with a polyclonal α–NikR antibody

Bacterial cultures of wild type and Δ*nikR* strains were grown to OD_600_ of 1.0–1.1 and split into 2 sub–cultures of 50 ml each that were treated with 500 μM nickel (ni+) or with the same volume of sterile water (ni−) for 20 minutes. Samples were crosslinked with 1% formaldehyde for 10 min at room temperature, then the reaction was stopped by treatment with 125 mM glycine for 10 min at room temperature, centrifuged 3900 g for 10 min at 4 °C and washed twice in 50 ml of cold 1X PBS followed by same steps of centrifugation. Samples were resuspended in 500 μl of TE (10 mM Tris, 1 mM EDTA; pH 8) with 2 mg/ml lysozyme solution, incubated 1 hr at 4 °C on a rotation wheel, diluted with 500 μl of 2X sonication buffer (10 mM Tris pH 8, 1 mM EDTA pH 8, 200 mM NaCl, 0.2% Sodium deoxycholate, 0.2% Igepal, 0.2% SDS, 1.5% Triton X–100), sonicated with Bioruptor (Diagenode) at high power for 80 min (30 sec ON–30 sec OFF) and diluted with 200 μl of dilution buffer (10 mM Tris pH 8, 1 mM EDTA pH 8, 340 mM NaCl, 0.1% Sodium deoxycholate, 0.1% Igepal, 2.25% Triton X–100). After a centrifugation of 3400 g for 8 min at 4 °C, 50 μl of the surnatant were used for Input preparation, while 1.1 ml was incubated with 50 μl of recombinant protein G – sepharose (Life Technologies) pre–equilibrated in RIPA buffer (10 mM Tris pH 8, 1 mM EDTA pH 8, 140 mM NaCl, 0.1% Sodium deoxycholate, 0.1% Igepal, 1% Triton X–100) for 1 hr a 4 °C. The NikR antibody was added to the pre–cleared surnatants at a 1:200 dilution; after incubation at 4 °C for 16 hr on a rotation wheel 50 μl of protein G – sepharose beads pre–equilibrated in RIPA buffer were added and binding reaction was carried out for 3 hr at 4 °C on rotation. The beads were washed four times in cold RIPA buffer, twice in cold TE, resuspended in 100 μl of TE solution with 20 μg/mL RNase A, and incubated at 37 °C for 30 min. SDS was added at a final concentration of 0.5% and proteinase K at a final 50 μg/mL concentration, followed by 16 hr incubation at 37 °C and addition of the same amount of proteinase K. Surnatants were phenol–chloroform extracted twice, chloroform extracted twice and ethanol precipitated with the addition of 20 μg of glycogen (Sigma–Aldrich). Input samples were obtained from 50 μl of sonicated material, following the same procedures described for the IPs, starting from RNase incubation step.

### DNase I footprinting

The DNA probes were prepared as follows: 1 pmol of pGEM–P*ureA*, pGEM–*cpdB*, pGEM–*vdlC*, pGEM–P*fecD*, pGEM–P*hopW*, pGEM–P*mccB*, pGEM–P*hpn* and pGEM–P*hpn2* vectors were linearized with NcoI, while pGEM–P*hcpC*, pGEM–P*hopV*, pGEM–P*dvnA*, pGEM–*exsB*, pGEM–*dapD*, pGEM–*pcrA*, pGEM–P*nnr1*, pGEM–P*isoB* and pGEM–P*phbA* vectors were linearized with NdeI, dephosphorylated with calf intestinal phosphatase and labeled at the 5′ ends with 2 pmol of [γ^–32^P] ATP (6000 Ci/mmol; PerkinElmer) by using T4 polynucleotide kinase. The labeled DNA probe was further digested either with NdeI or NcoI and the products were separated by native polyacrylamide 4% gel electrophoresis, eluted and purified as previously described^55^. The binding reactions were carried out by using approximately 20 fmol of labeled probe and increasing concentrations of NikR protein (from 9.7 to 290 nM of the NikR tetramer) at room temperature for 15 min in a final volume of 50 μl in footprinting buffer with 300 ng of salmon sperm DNA (Invitrogen) as a nonspecific competitor. Afterwards, DNase I (0.075 U), diluted in footprinting buffer containing 10 mM CaCl_2_ and 2.5 mM MgCl_2_ was added to the reaction mixture (2 μl) and digestion was allowed to occur for 90 s. The reaction was stopped, purified and resuspended in formamide loading buffer; samples were denatured at 100 °C for 3 min, separated on 8 M urea –6% acrylamide sequencing gels in TBE buffer and autoradiographed; a modified G + A sequencing ladder protocol was employed to map the binding sites, all according to ref. 55.

### ChIP-sequencing

Illumina libraries were prepared, for each of the conditions and strains analysed either from 5 ng of the two biological replicates of immunoprecipitated-DNA (IPs) or from 5 ng of the two biological replicates of the Input-DNA (INPUT) following the Illumina TruSeq ChIP-seq DNA sample preparation protocol; then each library was sequenced on a GAIIx or MiSeq Illumina sequencer and 51 bp single stranded reads were produced.

### RNA-sequencing

Ribosomal RNAs were depleted starting from 1 μg of total RNA from each of the conditions analyzed by using the RiboZero Gram negative kit (Epicentre, Illumina). Strand specific RNA-seq libraries were prepared by using the ScriptSeq^TM^ v2 RNAseq library preparation kit (Epicentre, Illumina) starting from 50 ng of previously rRNA depleted RNA from each biological replicate and for all the conditions analyzed. Then each library was sequenced on a GAIIx or MiSeq Illumina sequencer and 76 bp reads were produced. Bam files are publicly available at Sequence Reads Archive (SRA) under accession number BioProject PRJNA313048.

### Reads mapping quality assessment

Bowtie 2 (v2.2.6) was used to align raw reads, produced from both ChIP and RNA sequencing experiments, to *H. pylori* G27 genome (RefSeq GCF_000021165.1). End-to-end mapping was performed and non-deterministic option was specified to force a single assignment of multi-mapping reads to the best scoring region (if present) or a random attribution in the case of regions with identical scores. High quality reads were then selected requiring: for uniquely mapping reads MAPQ (mapping quality) greater than 30 and alignment score greater than −10 in ChIP-seq or −15 in RNA-seq samples; for multi-mapping reads alignment score were set equal or greater than −10 for ChIP-seq or −15 for RNA-seq.

The quality of ChIP-Seq data was evaluated following ENCODE quality metrics^56^ and the numerical values obtained are provided in Supplementary Table S1. The cross-correlation analysis resulted in good NSC and RSC values. Moreover we obtained average PBC scores. For RNA-seq samples rRNA depletion, strand specificity and gene coverage were evaluated using BEDTools (v2.20.1) and SAMtools (v0.1.19) (see Supplementary Table S1).

### ChIP-seq analysis

Irreproducible Discovery Rate procedure (IDR v 2.0.2) following ENCODE guidelines, and using Homer (v4.7.2) as peak caller, was performed to measure sample reproducibility and to identify consistent peaks. Homer parameters were set according to the authors’ indication for IDR calculation (-P 1 -LP 1 -poisson 1), -L was set to three and the fragment length was manually specified according to the median length of the sequencing library distribution. The “Fold Change vs Control” column was selected as ranking column for IDR calculations. R package DiffBind (v1.12.3) was adopted, without background reads subtraction, to determine differential bindings among the tested conditions. Δ*nikR*-nickel pooled samples were used as input/background for all the other experimental conditions. Peaks were manually classified as “promoter peaks” if centered −150/+30 from a TSS, as “intragenic peaks” if centered inside annotated genes and more than 30 nt apart from a TSS, and “intergenic peaks” if centered in unannotated regions and located farther than 150 nt form a TSS. TSS annotation was obtained cross-mapping onto G27 genome the 50 nt sequence upstream the 26695 published list of TSS^20^ and manually verifying the correspondence of the loci.

### RNA-seq analysis

Strand specific reads overlapping to coding sequences for at least 50% of their length were considered to produce the raw-counts of each sample. The R package DESeq2 (v1.4.5) was then used to normalize the counts and to individuate differentially expressed features showing log2FC ≥ |1| and BH adjusted p-value lower than 0.01. Genes were annotated to the current version of *H. pylori* G27 RefSeq annotation (GCF_000021165.1) and Inparanoid v4.1 was adopted to obtain protein ortologues in the reference strain *H. pylori* 26695. Old *H. pylori* G27 annotation gene names, *H. pylori* 26695 ones and common gene names (if available) are reported in parallel to the last *H. pylori* G27 annotation to facilitate results comparison.

### Consensus sequence analysis

The newly validated NikR promotorial binding sites as well as the previously individuated ones (or their homologous regions in G27 strain if the original strain was different from HPG27) were used as input for the consensus analysis. Considering the previous reports that highlighted an A/T-rich pseudo-palindromic recognition sequence for HpNikR targeted promoters^10^,^22^,^25^, we adopted GLAM2 which is specialized in finding gapped motifs to individuate NikR binding sequence, using the default parameters of the tool.

### Availabiliy of data and materials

Raw data supporting the conclusions of this article are available in the Sequence Reads Archive under accession number BioProject PRJNA313048; additional datasets are available within the Supplementary Tables 1,2,3,4,5,6 associated with this article.

## Additional Information

**How to cite this article:** Vannini, A. *et al*. Comprehensive mapping of the *Helicobacter pylori* NikR regulon provides new insights in bacterial nickel responses. *Sci. Rep.* **7**, 45458; doi: 10.1038/srep45458 (2017).

**Publisher's note:** Springer Nature remains neutral with regard to jurisdictional claims in published maps and institutional affiliations.

## Supplementary Material

Supplementary Information

Supplementary Table S1

Supplementary Table S2

Supplementary Table S3

Supplementary Table S4

Supplementary Table S5

Supplementary Table S6
