# Supplementary material for: Comprehensive mapping of the Helicobacter pylori NikR regulon provides new insights in bacterial nickel responses
Source: Sci Rep. 2017 Apr 10;7:45458. doi: 10.1038/srep45458 (PMC5385501; doi:10.1038/srep45458)
Supplement: Supplementary Information [file srep45458-s1.pdf]

Comprehensive mapping of the *Helicobacter pylori* NikR regulon provides new insights in bacterial nickel responses

Andrea Vannini [1,3*] & Eva Pinatel[2*], Paolo Emidio Costantini[1], Simone Pelliciari[1], Davide Roncarati[1], Simone Puccio[2,4], Gianluca De Bellis[2], Clelia Peano[2§] and Alberto Danielli[1§]

[1] FaBiT - Department of Pharmacy and Biotechnology, University of Bologna, 40126, Bologna, Italy
[2] Institute of Biomedical Technologies, National Research Council, 20090 Segrate, Milan, Italy
[3] DIMES Department of Experimental, Diagnostic and Specialty Medicine, University of Bologna, 40138 Bologna, Italy
[4] Doctoral School of Molecular and Translational Medicine, University of Milan, 20090 Segrate, Italy

* equally contributed
§ co-corresponding

SUPPLEMENTARY INFORMATION

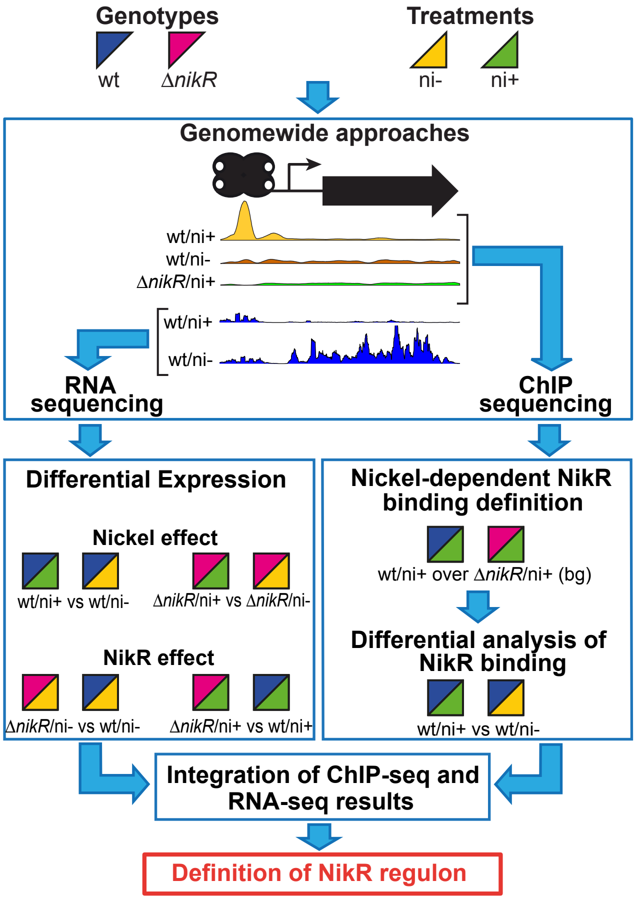

Fig S1. Schematic representation of the experimental design.
Bacterial cultures of the wt and Δ*nikR* mutant treated for 20 min with $NiSO_4$ 500 µM (ni+) or untreated (ni-) were used as starting material for ChIP and RNA sequencing. The effects of nickel treatment and *nikR* knockout were analysed to unravel direct and indirect effects in the NikR regulon through the integration of the two different datasets.

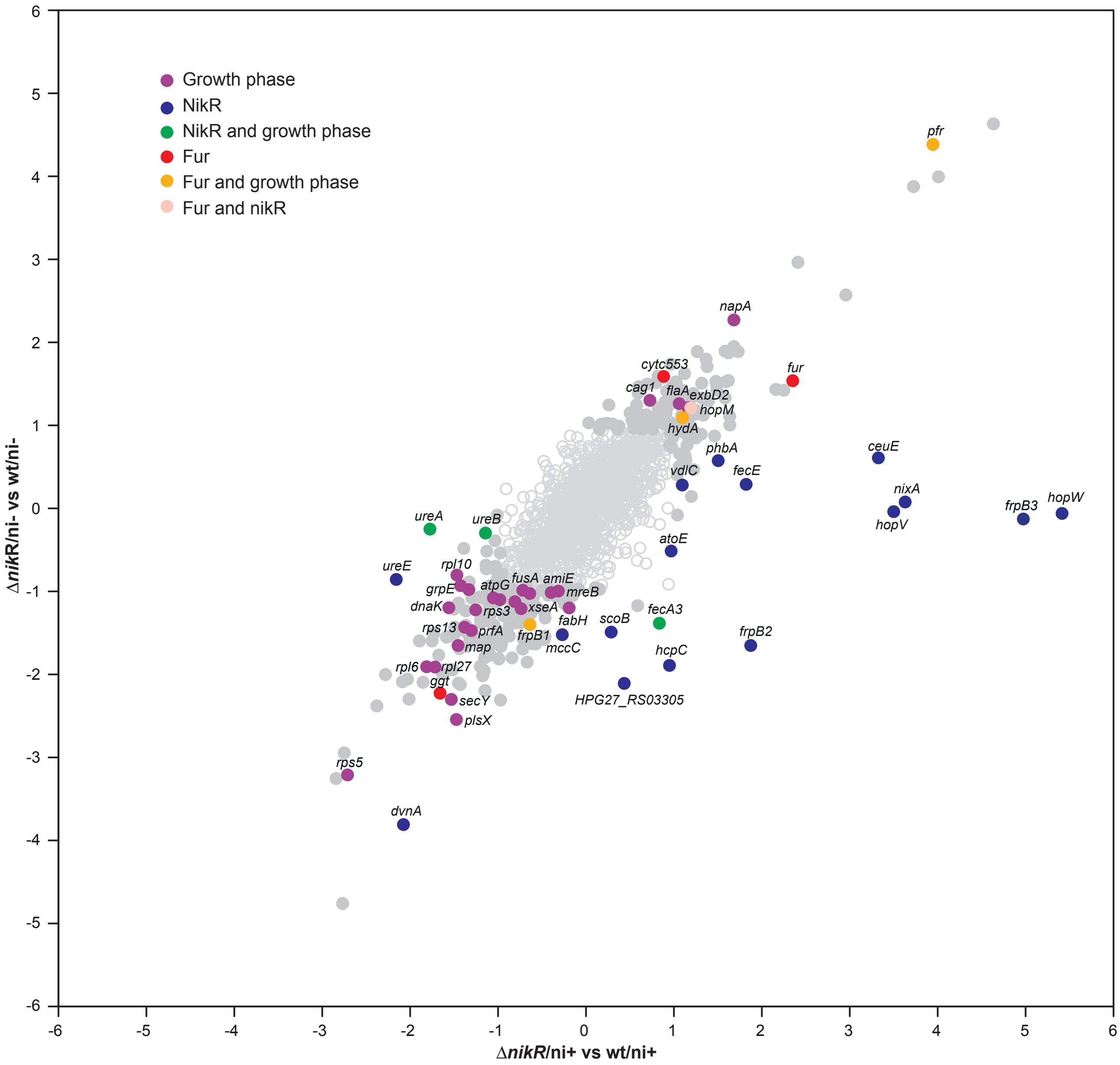

Fig S2. Effect of *nikR* deletion on *H. pylori* transcript levels.
RNA-seq derived log2FC values for Δ*nikR* vs wt genotypes are reported both in presence (x-axis) and absence (y-axis) of nickel treatment. Differentially expressed genes (log2FC ≥|1|; adj p <0.01) in at least one treatment are represented as filled circles; empty grey circles correspond to non-regulated genes. Analysis between the wt and Δ*nikR* strains treated with nickel (x-axis) outlined 194 DEGs. The upregulated genes include several apo–Fur repressed genes (red), together with the fur gene itself, and other genes reported to be induced in the late phase of bacterial growth (violet). Oppositely, several holo-Fur repressed genes and/or genes that are repressed in the stationary growth phase (violet/orange) resulted downregulated in this comparison, even if the wt and *ΔnikR* strains were grown in parallel to the same OD values (Supplementary Table S2). The same comparative analysis in the absence of nickel treatment (y-axis) supported this interpretation. In fact, out of the 261 DEGs identified, only a handful of nickel-responsive genes pinpointed in the wt strain were spotted, while most differentially expressed transcripts belong to stationary phase- and/or Fur-regulated genes (Supplementary Table S2).

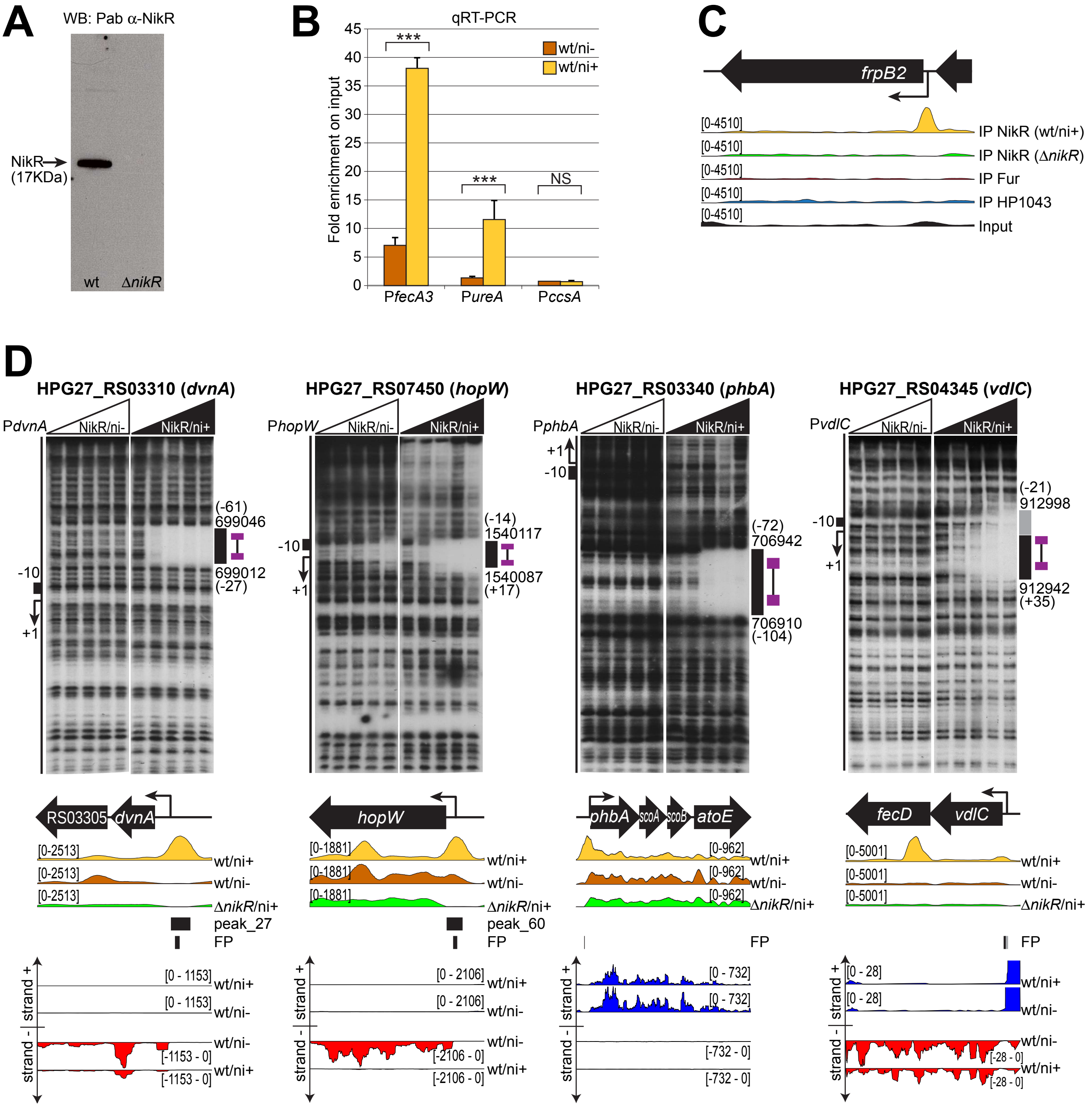

Fig S3. Validation of additional new NikR-regulated promoters by DNase I footprinting.

A) Western blot using the purified polyclonal antibody anti-NikR (1:1000) and the bacterial lysate of wt (left) or Δ*nikR* strains (right). B) Evaluation of the enrichment of the DNA regions encompassing P*fecA3*, P*ureA* (specific NikR targets) and P*ccsA* (non-specific target) by qRT-PCR on the IPs before the generation of the sequencing libraries, normalized on INPUT (chromatin sheared but not immunoprecipitated). C) ChIP-seq profiles of the *frpB2* genomic locus: using the anti-NikR antibody in the wt strain (wt/ni+), Δ*nikR* (negative control), performed with antisera specific for other transcriptional regulators (Fur or HP1043) or the profile of the INPUT control. D) DNase I footprinting of radiolabeled P*dvnA*, P*phbA* and P*vdlC* DNA probes with 0, 9.7, 29, 97 and 290 nM of the NikR tetramer and P*hopW* probe with 0, 0.53, 1.6, 4.9 and 14.7 nM of the NikR tetramers, without nickel (left side of each panel) or with the addition of 150 μM $NiSO_4$ (right side of each panel). Legends and symbols as in Fig. 2.

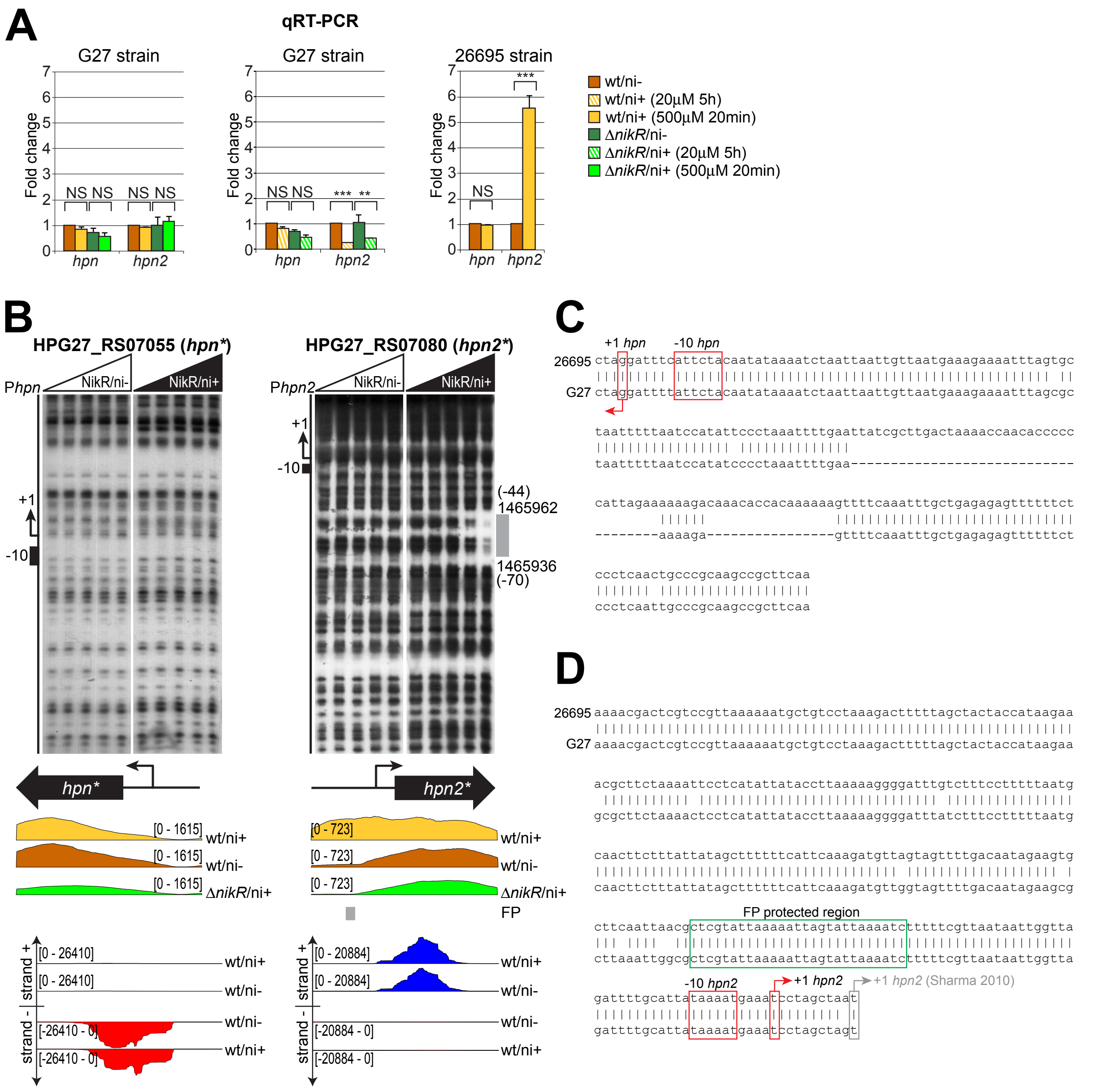

Fig S4. *hpn* and *hpn2* and the G27 NikR regulon.

A) *hpn* and *hpn2* gene expression levels are reported for wt and Δ*nikR* strain treated with $NiSO_4$ 500 μM for 20 min or 20 μM for 5h and untreated. Results show fold changes (mean±SD) relative to the wt/ni- condition. ΔCts were obtained after normalization on 16S rRNA levels and at least three biological replicates were performed. B) Footprinting of radiolabeled *hpn* and *hpn2* DNA probes mixed with 0, 8.7, 29 and 87 nM of NikR tetramers, without nickel (left side of each panel) or with the addition of 150 μM $NiSO_4$ (right side of each panel). Legends and symbols as in Fig. 2. Asterisks indicate a different reading frame considered for the *hpn* and *hpn2* genes, which are mis-annotated in the refseq GCF_000021165.1 annotation. Alignment of *hpn* (C) and *hpn2* (D) promoter regions in *H. pylori* 26695 (upper) and G27 (lower) strains, outline a significant deletion in the *hpn* promoter in strain G27.

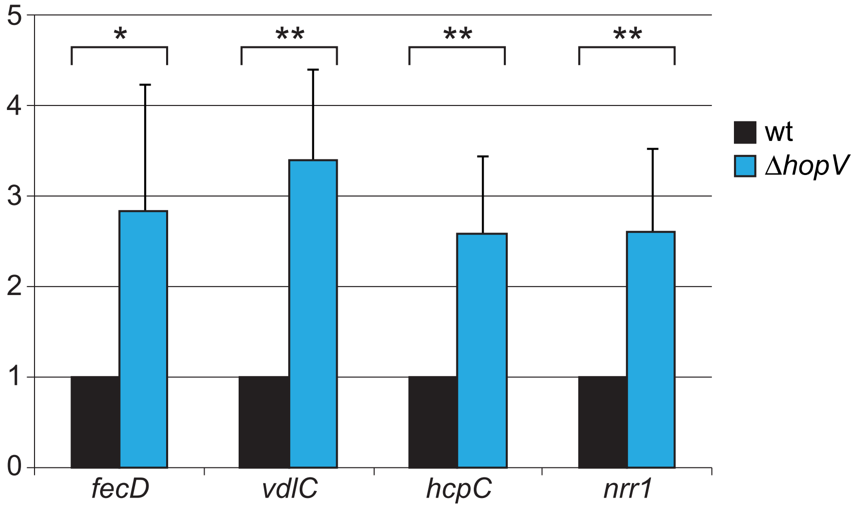

Fig S5. Functional analysis of *hopV*

Effect of *hopV* deletion on the expression of NikR-regulated genes: FC values of the *fecD*, *vdlC*, *hcpC* and *nrr1* transcripts in the Δ*hopV* mutant (cyan) vs. the wild type strain (black) are reported. qRT-PCR normalization and statistical analysis as in Fig. 1.

Figure S6. Original uncropped blots presented in Fig. 3

A Nrr1 RNA blot

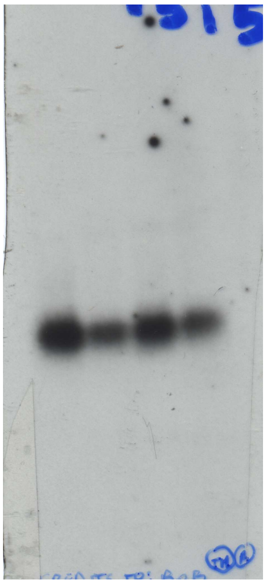

Ni- Ni+ Ni- Ni+

G27 wt replicate1 G27 wt replicate2

B Nrr2 primer extension

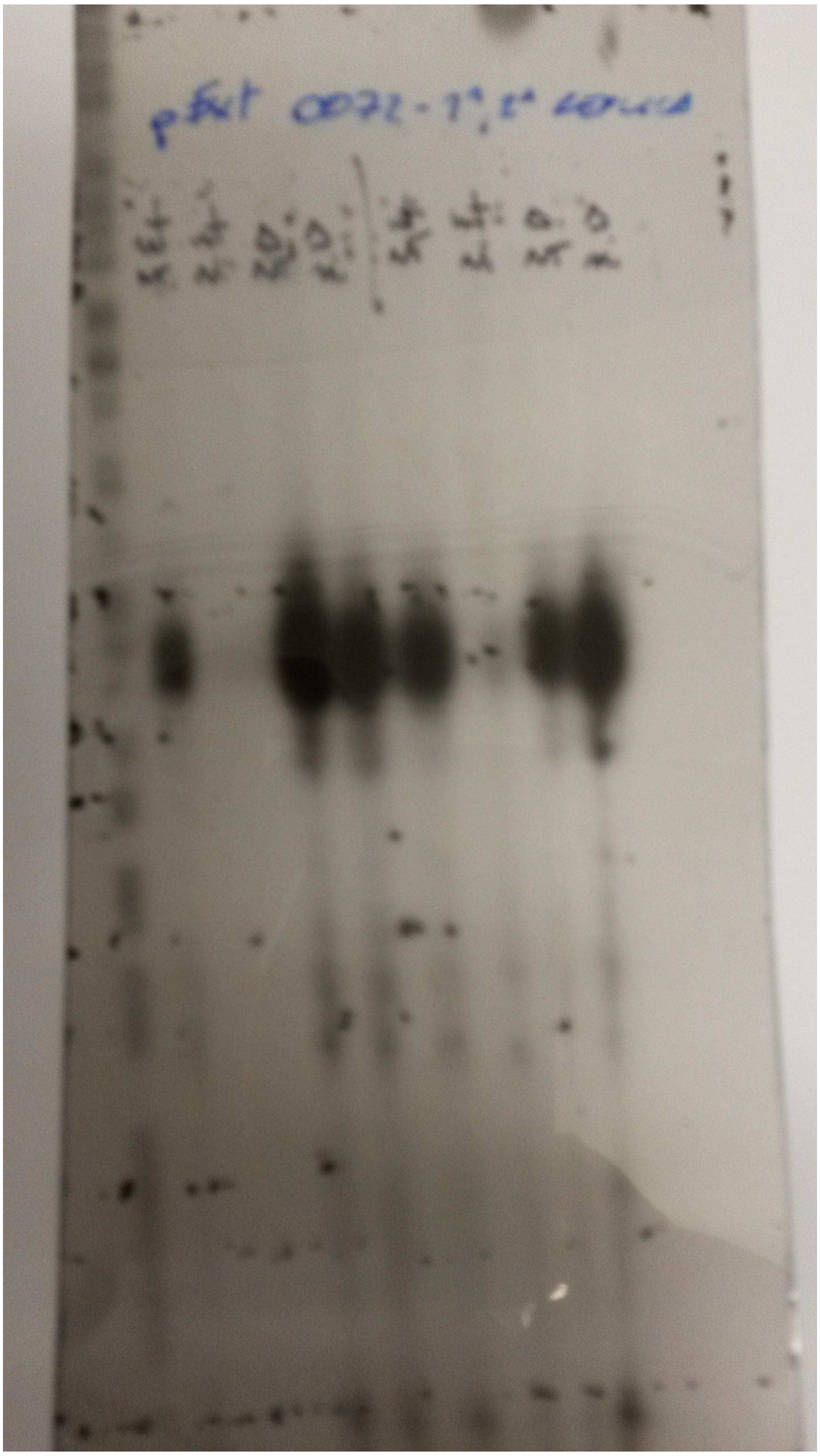

C IsoB RNA blot

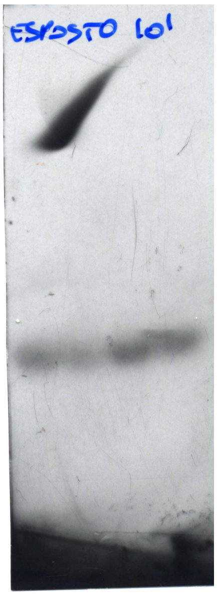

Ni- Ni+ Ni- Ni+

G27 wt delta *nikR*
