# Supplementary material for: Comprehensive mapping of the Helicobacter pylori NikR regulon provides new insights in bacterial nickel responses
Source: Sci Rep. 2017 Apr 10;7:45458. doi: 10.1038/srep45458 (PMC5385501; doi:10.1038/srep45458)
Supplement: Supplementary Table S4 [file srep45458-s5.doc]

**Comprehensive mapping of the Helicobacter pylori NikR regulon provides new insights in bacterial nickel responses**

Andrea Vannini & Eva Pinatel, Paolo Emidio Costantini, Simone Pelliciari, Davide Roncarati, Simone Puccio, Gianluca De Bellis, and Clelia Peano & Alberto Danielli

SUPPLEMENTARY TABLE 4

| **Name** | **Genotype or description** | **Source/reference** |
| --- | --- | --- |
| Strains |  |  |
| DH5α | λ^−^ φ80d*lac*ZΔM15 Δ*(lacZYA-argF)U169 recA1 endA hsdR17*(r_K_^−^ m_K_^−^) *supE44 thi-1 gyrA relA1* | (Hanahan 1983) |
| BL21 | F^−^*ompT hsdS*_B_(r_B_^−^ m_B_^−^) *gal dcm* (DE3) | ([Studier and Moffatt 1986](#_ENREF_37)) |
| G27 | Clinical isolate; wild–type parental strain | (Xiang, Censini et al. 1995) |
| G27 Δ*nikR* | G27 derivative; bp 88 to 417 of the *nikR* CDS replaced by a *km* cassette; Km^R^ | Pflock et al. 2005 |
| G27 Δ*hopV* | G27 derivative; with the *hopV* CDS (from bp -99 upstream the CDS to position 630 bp of the CDS) replaced by a *km* cassette; Km^R^ Cp^R^ | This study |
|  |  |  |
| Plasmids |  |  |
| pGEM–T Easy | Cloning vector, Ap^R^ | Promega |
| pGEM–P*ureA* | pGEM–T Easy derivative, carrying the 346 nt amplicon obtained with HP0072F and HP0072R oligonucleotides, encompassing the *ureA* promoter; Ap^R^ | This Study |
| pGEM–P*hcpC* | pGEM–T Easy derivative, carrying the 240 nt amplicon obtained with VS1098F and VS1098R oligonucleotides, encompassing the *hcpC* promoter; Ap^R^ | This Study |
| pGEM–P*dvnA* | pGEM–T Easy derivative, carrying the 327 nt amplicon obtained with VS682F and VS682R oligonucleotides, encompassing the *dvnA* promoter; Ap^R^ | This Study |
| pGEM–P*mccB* | pGEM–T Easy derivative, carrying the 257 nt amplicon obtained with VSPCD5F and VSPCD5R oligonucleotides, encompassing the *mccB* promoter; Ap^R^ | This Study |
| pGEM–P*hopV* | pGEM–T Easy derivative, carrying the 266 nt amplicon obtained with 1469F and 1469R oligonucleotides, encompassing the *hopV* promoter; Ap^R^ | This Study |
| pGEM–P*hopW* | pGEM–T Easy derivative, carrying the 401 nt amplicon obtained with HP1501 FW and HP1501 RV oligonucleotides, encompassing the *hopW* promoter; Ap^R^ | This Study |
| pGEM–P*phbA* | pGEM–T Easy derivative, carrying the 305 nt amplicon obtained with VS690F and VS690R oligonucleotides, encompassing the *phbA* promoter; Ap^R^ | This Study |
| pGEM–P*vdlC* | pGEM–T Easy derivative, carrying the 297 nt amplicon obtained with HP0890F and HP0890R oligonucleotides, encompassing the *vdlC* promoter; Ap^R^ | This Study |
| pGEM–P*hpn* | pGEM–T Easy derivative, carrying the 341 nt amplicon obtained with VSHpnF and VSHpnR oligonucleotides, encompassing the *hpn* promoter; Ap^R^ | This Study |
| pGEM–P*hpn2* | pGEM–T Easy derivative, carrying the 279 nt amplicon obtained with 1431DxD and 1431 DxS oligonucleotides, encompassing the *hpn2* promoter; Ap^R^ | This Study |
| pGEM–*dapD* | pGEM–T Easy derivative, carrying the 323 nt amplicon obtained with VS626F and VS626R oligonucleotides, encompassing a portion of the *dapD* CDS; Ap^R^ | This Study |
| pGEM–*exsB* | pGEM–T Easy derivative, carrying the 277 nt amplicon obtained with HP0639F and HP0639R oligonucleotides, encompassing a portion of *exsB* CDS; Ap^R^ | This Study |
| pGEM–*fecD* | pGEM–T Easy derivative, carrying the 241 nt amplicon obtained with HP0889F and HP0889R oligonucleotides, encompassing a portion of *fecD* CDS; Ap^R^ | This Study |
| pGEM– *pcrA* | pGEM–T Easy derivative, carrying the 244 nt amplicon obtained with VS1553F and VS1553R oligonucleotides, encompassing a portion of *pcrA* CDS; Ap^R^ | This Study |
| pGEM–P*nrr1* | pGEM–T Easy derivative, carrying the 244 nt amplicon obtained with VS1515F and VS1515R oligonucleotides, encompassing the ncRNA *nrr1* promoter; Ap^R^ | This Study |
| pGEM–P*isoB* | pGEM–T Easy derivative, carrying the 303 nt amplicon obtained with VSaapBF and VSaapBR oligonucleotides, encompassing the ncRNA *isoB* promoter; Ap^R^ | This Study |
| pBS–Δ*hopV* | pBluescript KS II derivative, carrying the XbaI/BamHI 507 bp amplicon obtained with VS1469UF and VS1469UR oligonucleotides, the BamHI/BamHI 1411 bp *km* cassette and the BamHI/XhoI 506 bp amplicon obtained with VS1469DF and VS1469DR oligonucleotides; Km^R^ Ap^R^ | This Study |
|  |  |  |
| Oligonucleotides | | |
| HP0072F | GTGTTTTTCCTTGAAGACATAAC | This Study |
| HP0072R | CAAAACAAAATTAAGGCATAATCAC | This Study |
| VS1098F | GGCTTACCCCTAAAGATTTAATTC | This Study |
| VS1098R | TTAGGGTCTTGCTCTGCCATTAAC | This Study |
| VS682F | CACCGCATAAAACCACTACACAAC | This Study |
| VS682R | CACGTGCCCCTCAAATTAAC | This Study |
| VSPCD5F | AACTTTGAATAATTACACTCTAAAATAG | This Study |
| VSPCD5R | GTTGGTATGGGCGGTAAG | This Study |
| 1469F | TTGCAGCGAGAAATCCAACG | This Study |
| 1469R | GCTAAAACACCCAAAGCCCC | This Study |
| HP1501 FW | GAGCGCCTAAAATTACGAG | This Study |
| HP1501 RV | CCCATCAAAAAACAAGTAGC | This Study |
| VS690F | GTGCATATCTGCTTGATTTTAGATCC | This Study |
| VS690R | TTTAAGCACGCAAACACCCATTTC | This Study |
| HP0890F | AAGTCGCATGCCTAGAGAGC | This Study |
| HP0890R | GCGATGATTGTATTTGAGGC | This Study |
| VSHpnF | TAAAATCAAAAACCAGAGACAACC | This Study |
| VSHpnR | TTAGCGCTAAATTTTCTTTCATTAAC | This Study |
| 1431DxD | GCTACTACCATAAGAAGCGCTT | This Study |
| 1431DxS | TTCATGGTGTGCCATGATGACT | This Study |
| VS626F | AGACAAAATCCTAAAAGAGAGCG | This Study |
| VS626R | AAATACCCTCTAAATTCAAACTCC | This Study |
| HP0639F | TAATCGCCGTGTTTGATCCT | This Study |
| HP0639R | GATTTTTTAGAGAATATCACCCGC | This Study |
| HP0889F | GCTCCGCTAGAAATCCCTAG | This Study |
| HP0889R | TGTTTGGAGGGGAGTCCTTG | This Study |
| VS1553R | CTAGTGAATGCAACATAATACAC | This Study |
| VS1553R | TGACCATTCACAAATCTAAAGG | This Study |
| VS1515F | AGTAAGGCCAATAACTTAAGCTC | This Study |
| VS1515R | TGTTTTTTGGACATAGTAATCCTTTG | This Study |
| VSaapBF | AACCCCTTTATCCCATCAATAGGC | This Study |
| VSaapBR | AAGCTTGCGTTTTTTTAAAGGGGA | This Study |
| 16S–RTF | GGAGTACGGTCGCAAGATTAAA | (Loh, Shaffer et al. 2011) |
| 16S–RTR | CTAGCGGATTCTCTCAATGTCAA | (Loh, Shaffer et al. 2011) |
| NikRL | ACGCATGTTTTATGCACCAC | De Reuse, 2011 |
| NikRTR | AGTTGCAAGCGTTGGATTTC | This Study |
| VSureRTF | TTGCATCAATTCAGCCGCAG | This Study |
| VSureRTR | TTGATGCTCCACTACGCTGG | This Study |
| NixRTF | CTTACATGCTAGGGGCAAAG | This Study |
| NixAR | CATAGGCGTTTTTGCCTTGT | De Reuse, 2011 |
| VS1512RF | TATGGGGCTAAAGCGGAAGC | This Study |
| VS1512RR | GCGTTGTTCCCGTCTCTGTA | This Study |
| VS1469RF | GATCTCTTTACTGATGCTCGTTGC | This Study |
| VS1469RR | ATGGTTTTTGGTTTTGTGGTAGGG | This Study |
| 1098RTF | GGGTGCTTTATTATCAAGGGCA | This Study |
| 1098RTF | GCTTTAGCGTAAAAGGAGGCG | This Study |
| VS682RTF | CCACCTCCTATATCTCTACCAAGC | This Study |
| VS682RTR | TGGTTAATGTGGCTGTAGCTAGTC | This Study |
| VS690RTF | GTTTGTGGATCGTCTATGAAAGCC | This Study |
| VS690RTR | TCTCGCATGTCAAACGACAAATAG | This Study |
| VSPCD5RF | AAATTTCCAATACCCCCACAACC | This Study |
| VSPCD5RR | CGAACAAAACTTTAAGAATCACCTG | This Study |
| 889RTF | CCCTGAAAGCACCAACGAAAG | This Study |
| 889RTR | GGCCAGCCTTGCTGTTTTG | This Study |
| VS1515RF | AGATTAGGGGGTAAATCACCTTCA | This Study |
| VS1515RR | GGGAAACCTTTGGCTTAATGGG | This Study |
| HpnRTF | CTAGCGCTGCAACAACCTTC | This Study |
| HpnRTR | CATGAAGAACAACACGGCGG | This Study |
| VShpn2RF | AATTAAAGGAGTCATCATGGCACAC | This Study |
| VShpn2RR | CTTGTTGCTCTGCTTGTTGCTC | This Study |
| VS1515R3 | ATAATACAAGGGCTTTTTACCATGT | This Study |
| VSaBRTR2 | TTAATGAAAAAGTTGATTAAAATGCTAAGT | This Study |
| VS0072p1 | CCATTATCACTCCAATTTTAATTCTC | This Study |
| VS890RF2 | GCATGCCTAGAGAGCGCATA | This Study |
| VS890RR2 | AAGGTGGCGGTTATCACTGG | This Study |
| VS1469UF | AAAAtctagaCGCTTTTACTACTTCAGTGC | This Study |
| VS1469UR | AAAAggatccAAAGTGCCTACGATCACG | This Study |
| VS1469DF | AAAAggatccAACTCAATTAAAAAACTAACC | This Study |
| VS1469DR | AAActcgagTGGGGAGTAAGAAATTGAG | This Study |
| PfecA3RTF | TTCACTAGAGATTGAGCAAA | This study |
| PfecA3RTR | GATAACGAACGCCTATTAAGT | This study |
| PureARTF | AACGCGCTTCTAATAACGCT | This study |
| PureARTR | GGTTGGATGTAATTGTAGCAATGTT | This study |
| VSp1461R | AACGCTATTTTAGGAGTTCATCAT | This study |
| VS1461R | GGGCATGAGCGCAAGAAAAG | This study |
